# Structural and Functional Elucidation of Yeast Lanosterol 14α-Demethylase in Complex with Agrochemical Antifungals

**DOI:** 10.1371/journal.pone.0167485

**Published:** 2016-12-01

**Authors:** Joel D. A. Tyndall, Manya Sabherwal, Alia A. Sagatova, Mikhail V. Keniya, Jacopo Negroni, Rajni K. Wilson, Matthew A. Woods, Klaus Tietjen, Brian C. Monk

**Affiliations:** 1 New Zealand’s National School of Pharmacy, University of Otago, Dunedin, New Zealand; 2 Sir John Walsh Research Institute, New Zealand’s National Centre for Dentistry, University of Otago, Dunedin, New Zealand; 3 Department of Oral Sciences, New Zealand’s National Centre for Dentistry, University of Otago, Dunedin, New Zealand; 4 Bayer SAS, Division Crop Science, Disease Control Research, Lyon, France; 5 Bayer AG, Division Crop Science, Disease Control Research, Monheim, Germany; Venenum Biodesign, UNITED STATES

## Abstract

Azole antifungals, known as demethylase inhibitors (DMIs), target sterol 14α-demethylase (CYP51) in the ergosterol biosynthetic pathway of fungal pathogens of both plants and humans. DMIs remain the treatment of choice in crop protection against a wide range of fungal phytopathogens that have the potential to reduce crop yields and threaten food security. We used a yeast membrane protein expression system to overexpress recombinant hexahistidine-tagged *S*. *cerevisiae* lanosterol 14α-demethylase and the Y140F or Y140H mutants of this enzyme as surrogates in order characterize interactions with DMIs. The whole-cell antifungal activity (MIC_50_ values) of both the *R*- and *S*-enantiomers of tebuconazole, prothioconazole (PTZ), prothioconazole-desthio, and oxo-prothioconazole (oxo-PTZ) as well as for fluquinconazole, prochloraz and a racemic mixture of difenoconazole were determined. *In vitro* binding studies with the affinity purified enzyme were used to show tight type II binding to the yeast enzyme for all compounds tested except PTZ and oxo-PTZ. High resolution X-ray crystal structures of ScErg11p6×His in complex with seven DMIs, including four enantiomers, reveal triazole-mediated coordination of all compounds and the specific orientation of compounds within the relatively hydrophobic binding site. Comparison with CYP51 structures from fungal pathogens including *Candida albicans*, *Candida glabrata* and *Aspergillus fumigatus* provides strong evidence for a highly conserved CYP51 structure including the drug binding site. The structures obtained using *S*. *cerevisiae* lanosterol 14α-demethylase in complex with these agrochemicals provide the basis for understanding the impact of mutations on azole susceptibility and a platform for the structure-directed design of the next-generation of DMIs.

## Introduction

Since the introduction in 1973 of the imidazole antifungal agent enilconazole (imazalil, chloramazole) and the triazole triadimefon, multiple generations of the azole antifungals [1–4] have underpinned global food security by preventing or treating a wide range of diseases in plants caused by fungal pathogens (for review see Parker *et al*.) [5]. Azoles are routinely used to treat diseases caused by phytopathogenic fungi including eyespot disease and powdery mildew in wheat and barley, black rust, headblight and septoria leaf blotch in wheat, leaf scald in cereals, leaf spot in sugar beet, powdery mildew on grapes, black sigatoka in bananas, apple scab, and mold on citrus fruit as well as the production of toxins. Without agrochemical intervention such diseases can substantially reduce crop yields, wipe out important crops, or result in crop spoilage due to rots or mycotoxins such as aflotoxins in peanuts. While numerous classes of antimycotics are employed in agriculture, such as the benzimidazoles, phenylamides, dicarboximides, anilinopyrimidines, quinone outside inhibitors and carboxylic amides, the triazole antifungals alone account for ~20% of the global market share for systemic fungicides. In the United Kingdom prothioconazole (PTZ), tebuconazole (TBZ) and epoxiconazole are the three most commonly used fungicides [6]. In addition, the azoles are well suited for use in agriculture, since in some cases they have the advantage of enhancing crop growth independent of their effects on phytopathogens [7]. The continuing success of azole antifungals has been attributed to their efficacy in the field and the slow evolution of azole resistance that has usually been countered by the development of more active triazole fungicides such as PTZ. This triazolinethione was introduced in 2002. Some problems have been associated with the agricultural use of azole antifungals, particularly in relation to human health. These include the development of antifungal resistance (reduced susceptibility) not only in the targeted phytopathogens but also in the ubiquitous and medically important pathogen *Aspergillus fumigatus* [8–13]. Endocrine disruption has also become a consideration. The imidazoles appear more potent than triazoles in eliciting possible side-effects that have been detected in animal tissue culture, such as inhibition of the conversion of progesterone to testosterone as well as anti-androgenic effects due to aromatase inhibition [14–16]. In a large study profiling the toxicities of environmental chemicals, it was found that the reproductive toxicity caused by individual azoles varies widely [17].

**Fig 1:**
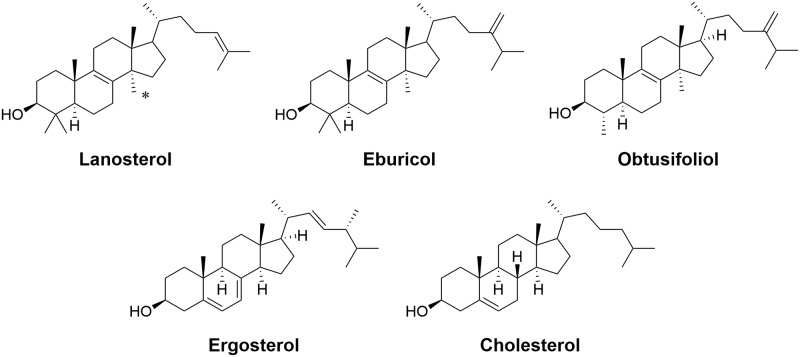
Substrates (lanosterol, eburicol and obtusifoliol) and products (ergosterol and cholesterol) of fungal pathogens as well as human and plant orthologues. * indicates methyl at 14 position.

Lanosterol 14α-demethylase (CYP51 or Erg11p in yeast and some fungal pathogens; sterol 14α-demethylase in fungal phytopathogens) is the primary target of the imidazole and triazole (azole) antifungal drugs [18], often referred to as demethylase inhibitors (DMIs). The CYP51 enzymes of fungal pathogens and yeast use eburicol or lanosterol as substrates to produce ergosterol while their orthologues in plants and humans use obtusifoliol and lanosterol, respectively, to produce plant sterols and cholesterol (Fig 1). In the absence of any X-ray structures of a fungal sterol 14α-demethylase, Mullins *et al*. (2011) proposed a structural rationale for the emergence of azole resistance associated with CYP51 mutations in the wheat blotch pathogen *Zymoseptoria tritici* (formerly *Mycosphaerella graminicola*) [19]. Homology models of the wild type protein and 13 variant proteins were generated by selectively combining the structures for *Mycobacterium tuberculosis* CYP51 plus a range of other CYP51 proteins as templates [19]. Modelling of the wild type and mutant binding pockets with the ligands TBZ, prochloraz (PRZ) and epoxiconazole implicated 85 amino acid residues in determining active site volume and azole binding, thereby suggesting a basis for the genetic selection of single and multiple mutations that led to reduced affinity for these antifungals.

We have determined the X-ray crystal structures of hexahistidine-tagged *Saccharomyces cerevisiae* lanosterol 14α-demethylase in complex with its substrate lanosterol, the pseudosubstrate estriol and the triazole drugs itraconazole (ITC), posaconazole (PCZ), fluconazole (FLC) and voriconazole (VCZ) [20–22]. These structures provided the first complete crystallographic analysis for a bitopic monospanning membrane protein, the first crystal structure for a fungal lanosterol 14α-demethylase and the first crystal structure to visualise the transmembrane domain of cytochrome 450, including its interaction with the enzyme’s catalytic domain. The yeast crystal structures identified the likely orientation of the enzyme in the lipid bilayer, the locations of a substrate entry channel and a putative product exit channel at the membrane surface that appears to contain zymosterol, a product requiring four subsequent steps in the ergosterol biosynthetic pathway. Previous studies of protein-protein interactions in the yeast sterol biosynthetic pathway [23] along with these structures are consistent with the idea that yeast lanosterol 14α-demethylase is part of a multienzyme complex named the ergosome, that includes NADPH-cytochrome P450 reductase, Erg24p (C-14 sterol reductase), and Erg25p-Erg27p (C-4 methyl sterol oxidase, C-3 sterol dehydrogenase and 3-keto sterol reductase respectively) in association with the Erg28 membrane scaffold protein. The lanosterol 14α-demethylase crystal structures have highlighted some other important features. The enzyme appears conformationally stable, particularly when in complex with a range of inhibitors, a substrate and a pseudosubstrate, with a total of 79 out of the enzyme’s 537 amino acids contributing to the surface of the haem-containing active site, the substrate entry channel and the product exit channel. Price *et al*. have used *S*. *cerevisiae* Erg11p as a template for an homology model of *Z*. *tritici* CYP51 [24]. In confirmation of biochemical observations on substrate specificity, the model predicted that the phytopathogen enzyme would prefer eburicol over lanosterol as a substrate, in contrast to the yeast enzyme’s preference for lanosterol. With the *Z*. *tritici* CYP51 homology model able to differentiate between substrates based on the yeast enzyme, the suggestion that up to 85 amino acids [19] contribute to the active site surface in the phytopathogen enzyme can now be significantly refined.

**Fig 2:**
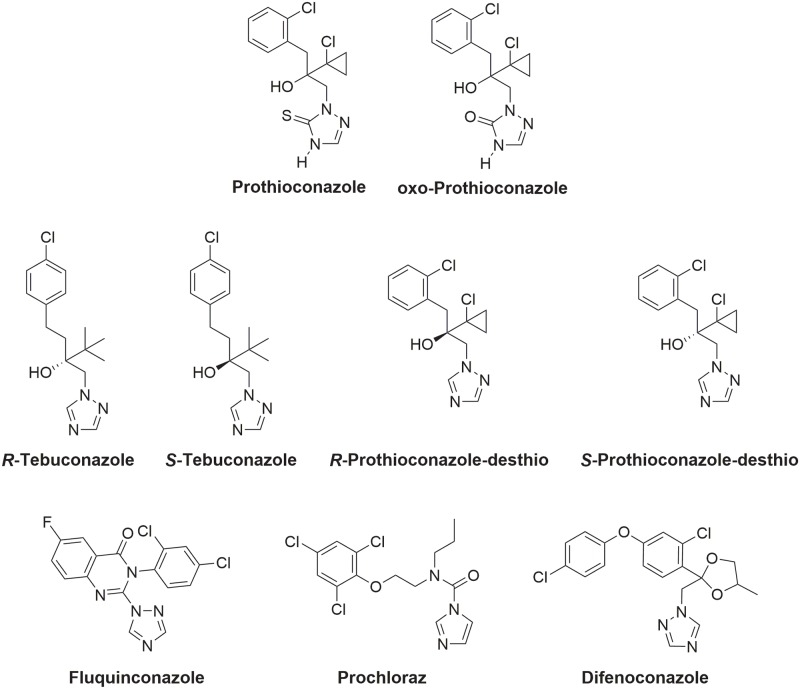
Chemical structures of investigated inhibitors.

In order to better understanding the activity of DMI agrochemicals, we have determined how selected antifungals interact with their target enzyme using *S*. *cerevisiae* lanosterol 14α-demethylase as a surrogate. Using recombinant *S*. *cerevisiae* lanosterol 14α-demethylase (ScErg11p6×His; wildtype) or the Y140F or Y140H mutants of this enzyme [25] we have determined the whole cell antifungal activity (MIC_50_ values) of the *R*- and *S*- enantiomers of TBZ, PTZ, prothioconazole-desthio (DPZ), oxo-prothioconazole (oxo-PTZ) as well as for fluquinconazole (FQZ), PRZ and a racemic mixture of difenoconazole (DFC, Fig 2). *In vitro* binding studies with the affinity purified enzyme showed tight type II binding for all compounds tested, apart from PTZ and oxo-PTZ. We have also determined seven high resolution X-ray crystal structures of ScErg11p6×His in complex with *R*- and *S*-TBZ, *R*- and *S*-DPZ, FQZ, PRZ and DFC. Until crystal structures are obtained for the sterol 14α-demethylases of individual phytopathogens, the structures of *S*. *cerevisiae* lanosterol 14α-demethylase in complex with these agrochemicals and with azoles used in medicine will provide a basis for the structure-directed discovery and design of next-generation DMIs.

## Results

### X-ray crystallographic analysis of phytopathogen CYP51 inhibitors in complex with ScErg11p6×His

Crystal structures of the full length *S*. *cerevisiae* lanosterol 14α-demethylase (ScErg11p6×His) in complex with *S*-TBZ (PDB ID:5EAB), *R*-TBZ (PDB ID:5EAC), *S*-DPZ (PDB ID:5EAD), *R*-DPZ PDB ID:5EAE), FQZ (PDB ID:5EAF), PRZ (PDB ID:5EAG), and all four stereoisomers of DFC (PDB ID:5EAH) were determined at resolutions from 2.65 to 2.00 Å. Data collection and refinement statistics are given in S1 Table. All complexes were crystallised in the space group P 1 2_1_1, with a single ScErg11p6×His-ligand complex in the asymmetric unit, with the exception of the ScErg11p6×His -FQZ complex, which was crystallised in space group P1 with two protomers found in the asymmetric unit. Comparison of the ScErg11p6×His-FQZ complex with the others shows an almost identical protein structure, including the transmembrane and amphipathic helices. All ligand-protein complexes were isomorphous with the original ScErg11p6×His lanosterol complex (PDB ID:4LXJ) used for molecular replacement [20].

### *S-* & *R*-Tebuconazole (TBZ)

**Fig 3:**
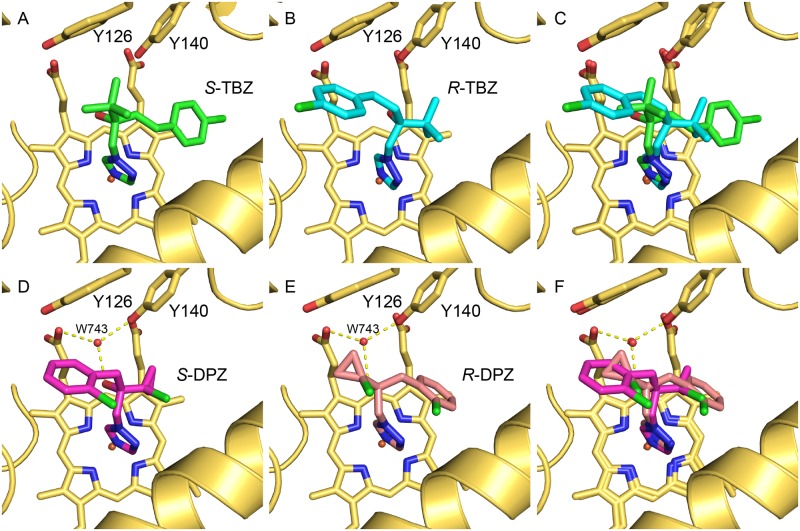
Phytopathogen inhibitors complexed in the active site of ScERG11p6×His. The structures reveal the active site binding orientation of the enantiomers (A) *S*-TBZ (green carbons, PDB ID:5EAB), (B) *R*-TBZ (cyan carbons, PDB ID:5EAC), and (C) the superimposition of *S*-TBZ and *R*-TBZ, the active site orientation of the enantiomers (D) *S*-DPZ (magenta carbons, PDB ID:5EAD), (E) *R*-DPZ (salmon carbons, PDB ID:5EAE), and (F) the superimposition of *S*-DPZ and *R*-DPZ. The heme cofactor and selected residues (Y126 and Y140) are shown as sticks. Water-mediated (w743, red sphere) hydrogen bonds are shown as yellow dashed lines. Helix I is shown as a yellow ribbon at the bottom right of each panel. Nitrogen atoms are blue, oxygen red and chlorine green.

Both *S-* and *R-*TBZ bind in the active site via a coordinate bond between the N3 nitrogen of the triazole and the iron of the heme cofactor (Fig 3A & 3B, S1 Fig). The hydroxyl group of both enantiomers point in the same direction toward Y126 and Y140. The position of these hydroxyl groups differs by 1.6 Å due to the large 4-chlorophenethyl group of *S*-TBZ (bounded by G310 in helix I and I139 in the loop joining helices B and C; Fig 3C) projecting in the active site to the position occupied by the disubstituted phenyl ring of FLC (PDB ID:4WMZ) [21]. The smaller tertiary butyl group of *S*-TBZ is positioned towards the substrate entry channel. The 4-chorophenethyl substituent of the less active *R* enantiomer (see below) is also positioned towards the entry channel i.e. in the opposite position to the *S* enantiomer (Fig 3B). The 4-chorophenethyl substituent of the *R* enantiomer is less well defined due to the additional space in this region (S1 Fig). The *tert*-butyl group occupies the cavity distal to the entry channel deep in the active site. The central quaternary carbons of the enantiomers are approximately 1 Å apart, with *R*-TBZ projecting deeper into the active site cavity which easily accommodates the smaller *tert*-butyl substituent (Fig 3C). The key water 743 identified in the wild type FLC structure [21, 22] is not seen in either TBZ structure. This may be due to the lower resolution of this structure but could easily be accommodated.

### *S*- & *R*-Prothioconazole-desthio (DPZ)

Both *S-* and *R*-DPZ were crystallised with ScErg11p6×His and were found to be unambiguously present in the active site (S1 Fig). Both enantiomers coordinate to the heme iron via N3 nitrogen of the triazole (Fig 3). The tertiary hydroxyl group of both enantiomers project towards the propionate of the heme and form water-mediated (water 743) hydrogen bonds to the heme as well as the phenolic hydroxyl of Y140. The 2-chlorophenyl ring of the *R* enantiomer occupies the deep cavity similar to FLC with the chlorocyclopropyl group occupying the larger cavity towards the entry channel. Conversely, the chlorocyclopropyl group of the more active *S* enantiomer (see below) is situated in the deep cavity distal to the entry channel with the 2-chlorophenyl ring occupying the cavity towards the entry channel and binds in the same plane as the non-coordinating triazole of FLC in the wild type structure [21]. The 2-chloro substituent of the phenyl ring is in close proximity to the phenyl ring of Phe236, causing it to move away from the inhibitor compared with the other enantiomer. A water molecule (802) is present between F384 and the chlorocyclopropyl group of the *R* enantiomer. This is displaced by the phenyl ring in the *S* enantiomer complex. Consequently, F384 and Y126 are oriented closer to the hydrophobic ring of the ligand. Attempts were made to crystallise PTZ and oxo-PTZ but crystals could not be obtained.

### Fluquinconazole (FQZ)

**Fig 4:**
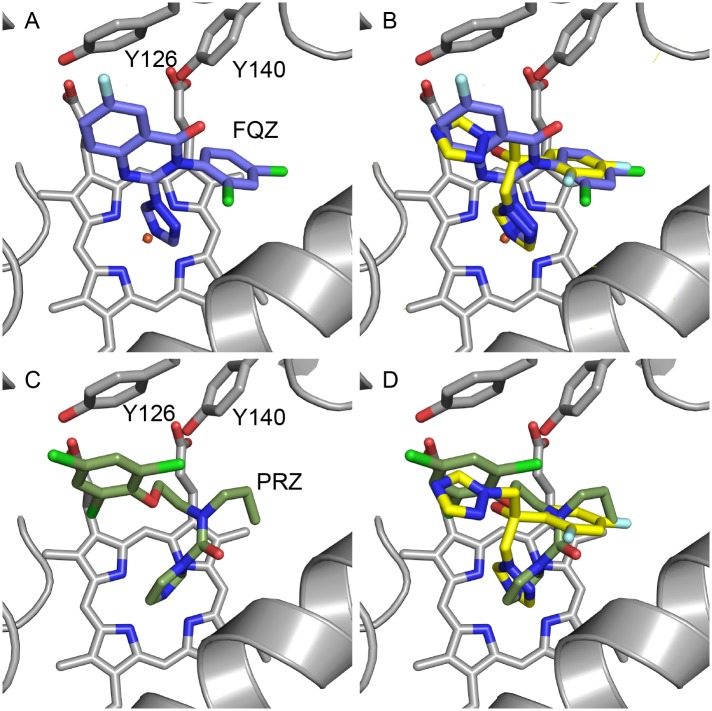
Phytopathogen inhibitors complexed in the active site of ScERG11p6×His. The structures reveal the binding orientation of (A) FQZ (purple carbons, PDB ID:5EAF), (B) the superimposition of FQZ with FLC (yellow carbons, PDB ID:4WMZ), the binding orientation of (C) PRZ (olive carbons, PDB ID:5EAG) and (D) the superimposition of PRZ with FLC (yellow carbons). The heme cofactor and selected residues (Y126 and Y140) are shown as sticks. Helix I is shown as a grey ribbon at bottom right of each panel. Nitrogen atoms are blue, oxygen red, chlorine green and fluorine pale blue.

FQZ binds unambiguously in the active site via a coordinate bond between the N3 of the triazole ring and the iron of the heme cofactor (Fig 4A; data in S1 Fig). The dichlorophenyl ring binds deep in the active site adjacent to G310 in a similar manner to FLC (Fig 4B). The rigid quinazolin-4(3H)-one ring projects towards the entry channel in an orientation with the aromatic ring perpendicular to the non-coordinating azole of FLC. No water (743/843) is seen adjacent to Y140. Y126 is shifted away compared to the FLC structure to accommodate the rigid and bulky ring system with a close contact between the fluoro substituent of the fungicide and the phenolic ring. The N1 nitrogen of the quinazolin-4(3H)-one ring is in close proximity to Cδ1 of L380, the carbonyl oxygen is close to Cγ2 of T130 and the coordinating triazole abuts G310 as seen previously with *S*-TBZ.

There are two protomers in the asymmetric unit and the interface between the two protomers includes direct hydrogen bonding interactions between the phenolic hydroxyl of Y341 and the main chain carbonyl oxygen of E418’ with a salt bridge between E342 and K107’. There are further water mediated hydrogen bonds between E339 Oε2 and K107’ Nζ. A limited number of water molecules were modelled into the structure of ScErg11p6×His in complex with FQZ due to the relatively low resolution of this structure. There is a van der Waals interaction between D335 and V441’. The amphipathic helix at the N-terminus lies approximately perpendicular to helix G (249–274) of an adjacent symmetry-related molecule and forms a salt bridge between E13 and K270” Nζ as well as a hydrogen bond with the Q302” N of helix I. Further interactions occur between N16 Nδ2 and Q302” Oε1. The amphipathic N-terminus of ScErg11p6×His in complex with lanosterol shows identical interactions between E13 and K270” and N16 with Q302, the interaction between Y341 and E415” in the catalytic domain was maintained but the interactions between E342 and E339 with K107” were not found. These data therefore do not exclude the possibility that crystal contacts may affect the orientation of the N-terminal membrane associated helix of ScErg11p6×His.

### Prochloraz (PRZ)

PRZ differs chemically from other inhibitors tested as it coordinates to the heme iron via the N3 nitrogen of an imidazole ring compared with a triazole ring. The plane of the amide group is approximately 60° to that of the imidazole ring and the carbonyl oxygen is in close proximity to G314 of helix I (Fig 4C). The *n*-propyl group occupies part of the deep hydrophobic pocket lined by I139 in the loop between helices B and C, with the acyclic nitrogen in a similar position to that of the ipso carbon of the dichlorophenyl ring of FLC (Fig 4D). This restricted conformation pushes the ethyl linker into the space near water 743 (PDB ID:4WMZ) with the second carbon being 3.3 Å from the phenolic hydroxyl of Y140, thereby excluding any water mediated hydrogen bonding. The 2-chloro substituent is in close proximity to both Cδ1 of L380 and Cγ2 of L383, while the phenyl ring pushes Y126 away from the active site into a position approaching a pi stacking interaction.

### Difenoconazole (DFC)

**Fig 5:**
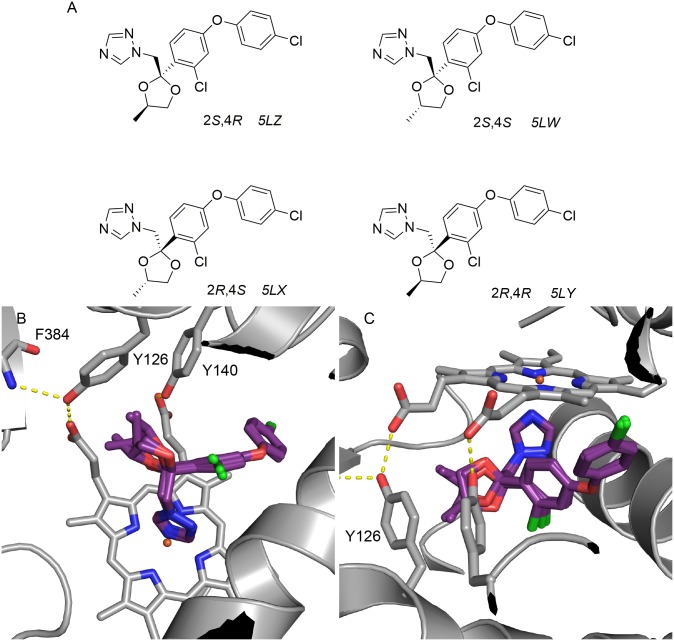
Stereochemistry and binding of the plant pathogen CYP51 inhibitor Difenoconazole. (A) Stereoisomers of DFC, (**2*S*,4*R***) - 1-(((2*S*,4*R*)-2-(2-chloro-4-(4-chlorophenoxy)phenyl)-4-methyl-1,3-dioxolan-2-yl)methyl)-1*H*-1,2,4-triazole; (**2*S*,4*S***) 1-(((2*S*,4*S*)-2-(2-chloro-4-(4-chlorophenoxy)phenyl)-4-methyl-1,3-dioxolan-2-yl)methyl)-1*H*-1,2,4-triazole; (**2*R*,4*S***) 1-(((2*R*,4*S*)-2-(2-chloro-4-(4-chlorophenoxy)phenyl)-4-methyl-1,3-dioxolan-2-yl)methyl)-1*H*-1,2,4-triazole; (**2*R*,4*R***) 1-(((2R,4R)-2-(2-chloro-4-(4-chlorophenoxy)phenyl)-4-methyl-1,3-dioxolan-2-yl)methyl)-1H-1,2,4-triazole. (B) Orientation of DFC stereoisomers (purple carbons, PDB ID:5EAH) bound within the active site of ScErg11p6×His. The heme cofactor and selected residues (Y126, Y140 and F384) are shown as sticks. Hydrogen bonds are shown as yellow dashed lines. Helix I is shown as a grey ribbon at bottom right. (C) Rotated view showing the projection of the 4-methyl substituent towards either Y126 or the heme cofactor. Nitrogen atoms are blue, oxygen red and chlorine green.

DFC was purchased as the mixture of all four stereoisomers and used without separation for the crystallisation experiment (Fig 5). Unambiguous density for the majority of the ligand was observed upon molecular replacement (data in S1 Fig). All four isomers were modelled and refined using occupancy refinement. Stereoisomers refined to occupancies of 30% (*2S*,*4R*), 29% (*2S*,*4S*), 0% (*2R*,*4S*) and 41% (*2R*,*4R*) respectively, suggesting that the *2R*,*4S* isomer may not be present whereas the other three each possess approximately one third occupancy. Overall, all isomers bind within the active site in a similar fashion. The triazole coordinates to the heme iron as expected, the substituted phenoxyphenyl group binds deep within the active site cavity between L307 in helix I, K151 in helix C and the heme methyl group. This is the largest moiety of any crystallised CYP51 inhibitor to occupy this cavity.

The differences between isomers are centred around the 1,3-dioxalane ring. Both the 2*S* isomers project the methyl towards Y126 but away from the heme whereas the 2*R* isomers have the methyl group projecting towards the heme (Fig 4C downwards and upwards respectively). Y126 forms a hydrogen bond with the heme propionate as well as the amide nitrogen of F384. Like the dioxolane moiety in ITC [20], the 4-methyl-1,3-dioxolane ring of DFC occupies the position where the key water 743 is found in the presence of FLC, *R*- and *S*-DPZ. The residue M509 projects into the binding site adjacent to H381 when compared to other structures, indicating a degree of flexibility around this position only previously seen in the ScErg11p6×His-VRC structure (PDB ID:5HS1) [22]. The elasticity at this position is generally dependent on the size of the inhibitor, with larger medium and long chain azoles occupying the space.

### Susceptibilities to azole inhibitors

The susceptibility to azole compounds of *S*. *cerevisiae* AD3Δ strains overexpressing Erg11p6×His wild type and Y140F/H, but with the native *ERG11* deleted, as well as the host strain (AD2Δ) which retained the wild type ERG11 (S2 Table), were measured as MIC_50_ values (Table 1). MIC_50_ values for the four sets of enantiomers reveal the more active inhibitors to be *S*-TBZ, *S*-PTZ, *S*-DPZ with the *S* and *R* enantiomers of oxo-PTZ [26] being weakly equipotent. PTZ has been reported to be a prodrug for DPZ [27]. We observed 8.7 fold (0.64 ± 0.11 μM) and 11.9 fold (0.87 ± 0.06 μM) reduction in susceptibility to *S*-TBZ in the Y140F and Y140H mutant strains respectively. There was a similar but less dramatic trend for the less active *R*-TBZ. There was a 3.3 fold (0.28 ± 0.02 μM) and a 4.3 fold (1.2 ± 0.2 μM) reduction in susceptibility to *S*-PTZ in the Y140F and Y140H mutant strains respectively. There was only a 2 fold reduction in susceptibility to *R*-PTZ in the Y140H mutant strain with no change seen in the Y140F strain. The PTZ metabolite, *S*-DPZ produced a 4.6 fold (0.073 ± 0.016 μM) and 3 fold (0.048 ± 0.009 μM) reduction is susceptibility against the Y140F and Y140H mutant strains respectively. A 2.3 fold reduction was seen for the less active *R*-DPZ against both mutant strains. The weak inhibitor oxo-PTZ shows no difference in susceptibilities between enantiomers. FQZ shows a similar potency (0.40 ± 0.05 μM) to the prodrug *S*-PTZ. Again we observed 8.3 fold (3.3 ± 0.7 μM) and 4 fold (1.6 ± 0.5 μM) reduction in susceptibility to FQZ in the Y140F and Y140H mutant strains respectively. PRZ is less active (1.9 ± 0.5 μM) with reduced susceptibility (2.3 fold and 3.5 fold) to Y140F and Y140H mutant strains respectively. DFC is the second most potent compound in our assay (behind *S*-DPZ) but also shows 5.2 fold (0.243 ± 0.003 μM) and 5 fold (0.237 ± 0.003 μM) reduced susceptibility against the Y140F and Y140H mutant strains respectively. All compounds showed significantly lower MIC_50_ values when tested with the AD2Δ strain expressing the native Erg11p, which led to higher fold differences in susceptibility and showed that the overexpressed enzyme was functional.

**Table 1:** MIC_50_ data for compounds against *Saccharomyces cerevisiae* strains.

Compound	MIC_50_ (μM)			
	AD2Δ	AD3Δ	Y140F	Y140H
*S*-Tebuconazole	0.0197 ± 0.0003	0.073 ± 0.015	0.64 ± 0.11	0.87 ± 0.06
*R*-Tebuconazole	0.12 ± 0.0	1.1 ± 0.3	2.0 ± 0.2	5.2 ± 1.3
*S*-Prothioconazole	0.077 ± 0.003	0.28 ± 0.02	0.93 ± 0.0	1.2 ± 0.2
*R*-Prothioconazole	5.2 ± 1.3	16 ± 3	16 ± 4	31 ± 4
*S*-Prothioconazole-desthio	0.006 ± 0.001	0.0157 ± 0.0003	0.073 ± 0.016	0.048 ± 0.009
*R*-Prothioconazole-desthio	0.47 ± 0.05	3.2 ± 0.2	7.5 ± 0.0	7.5 ± 0.0
*S*-Oxo-prothioconazole	75.0 ± 0.0	84 ± 3	87 ± 7	97 ± 3
*R*-Oxo-prothioconazole	75.0 ± 3.0	80 ± 2	87 ± 7	95 ± 5
Fluquinconazole	0.17 ± 0.02	0.40 ± 0.05	3.3 ± 0.7	1.6 ± 0.5
Prochloraz	0.25 ± 0.02	1.9 ± 0.5	4.3 ± 0.8	6.7 ± 0.7
Difenoconazole	0.0147 ± 0.0003	0.047 ± 0.009	0.243 ± 0.003	0.237 ± 0.003

Values are the mean for 3 separate clones of each strain using data obtained in triplicate measurements from at least 3 different experiments (Standard error is shown).

**AD2Δ** host with native *ERG11* retained.

**AD3Δ** - ScErg11p6×His overexpressed from *PDR5* locus, native *ERG11* deleted.

**Y140F** - ScErg11p6×His Y140F overexpressed from *PDR5* locus, native *ERG11* deleted.

**Y140H** –ScErg11p6×His Y140H overexpressed from *PDR5* locus, native *ERG11* deleted.

### Type II binding to affinity purified ScErg11p6×His

**Fig 6:**
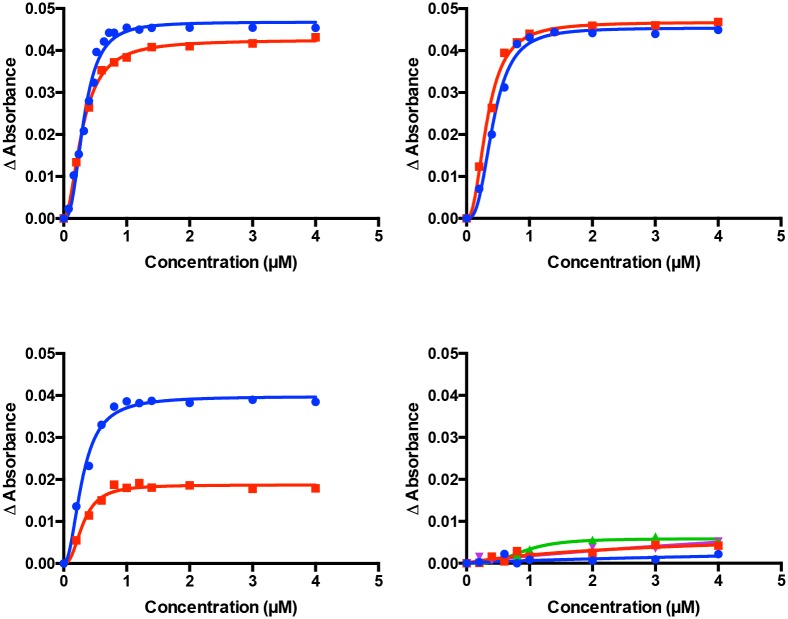
Azole inhibitor binding to ScErg11p6×His. Binding saturation curves for (A) *S*-TBZ (blue) and *R*-TBZ (red), (B) *S*-DTZ (blue) and *R*-DTZ (red), (C) PRZ (blue) and FQZ (red), and (D) *S-* and *R*-PTZ in blue and red; with *S* and *R*-oxo-PTZ in green and purple respectively showing no binding. Experiments were run at a minimum in duplicate.

The absolute absorbance spectra for Ni-NTA affinity and SEC purified ScErg11p6×His showed a Soret peak at 417 nm [21]. Type II azole binding is characterised by a shift of the Soret peak from 417 nm to 421–424 nm in ScErg11p6×His and other cytochrome P450s. The type II difference spectra were used to detect and quantitate binding of the inhibitors *S*-TBZ, *R*-TBZ, *S*-PTZ, *R*-PTZ, *S*-DPZ, *R*-DPZ, *S-*oxo-PTZ and *R*-oxo-PTZ, FQZ, and PRZ using Ni-NTA affinity purified wild type ScErg11p6×His preparations at 1 μM. Spectral shifts, [Azole]_0.5_ values, Hill coefficients as well as Type II difference spectra binding curves are shown in S3 Table (selected spectra shown in S2 Fig) and Fig 6, respectively. Equivalent spectral shifts from 417 nm to ~421 nm were obtained for enantiomers of TBZ and DPZ as well as for FQZ and PRZ. The absence of a spectral shift for enantiomers of PTZ or oxo-PTZ indicated there is no azole coordination at the heme iron. This was confirmed in the type II binding experiments. All other compounds showed type II binding with [Azole]_0.5_ values at ~ 0.4 μM in the presence of 1 μM functional ScErg11p6×His and K_d_ values in the low nanomolar range indicating tight binding. These results are comparable to a previous study which determined K_d_ values of DPZ and PTZ against *Candida albicans* CYP51 which showed tight type II binding (K_d_ 36.4 ± 10.7 nM) for the former, with the latter producing only a weak type I difference spectra (K_d_ 6.3 ± 1.5 μM) [27]. TBZ, DPZ, FQZ and PRZ were all shown to be non-competitive inhibitors of ScErg11p6×His as expected. However, compared with the competitive binding on *M*. *graminicola* CYP51 shown by Parker et al., PTZ showed no binding (S3 Table).

## Discussion

To better understand how antifungal CYP51 inhibitors of phytopathogens work, we have determined seven X-ray crystal structures using the surrogate enzyme ScErg11p6×His. These complexes reveal the specific binding orientation of individual enantiomers of two azole agrochemicals that are administered in the field as racemic mixtures (*S/R*-TBZ and *S/R*-DPZ) along with FQZ, PRZ and the racemic mixture of four stereoisomers of DFC. Biochemical studies have measured azole binding to the affinity purified enzyme and identified *S*-TBZ and *S*-DPZ as the more active enantiomers within the set of molecules tested against *S*. *cerevisiae* cells. There are currently no published structures for any fungal phytopathogens. However, the structures of other fungal pathogen CYP51s, including full-length *Candida glabrata* (PDB ID: 5JLC), full-length *Candida albicans* (Keniya, unpublished) as well as the N-terminally truncated *Aspergillus fumigatus* enzyme [28] provide strong evidence that the CYP51 structure is highly conserved across all fungal pathogens.

The triazolinethione fungicide PTZ was released in 2002 and has been previously shown to bind *Z*. *tritici* (*M*. *graminicola*) CYP51 via a mechanism different to other azoles such as epoxiconazole, TBZ, triadimenol [29] and DPZ [27]. Our studies confirm that enantiomers of DPZ but not enantiomers of PTZ, or oxo-PTZ [26], give type II binding to ScErg11p6×His. Our whole cell MIC_50_ data for both *S*-PTZ and *R*-PTZ reveal that both compounds are active against yeast. The *S*-enantiomers of both PTZ and DPZ were found to be the more active enantiomers [57 fold and 200 fold more active over the *R* enantiomers respectively (MIC_50_ values for ADΔ3 strains)]. Differences in activity between *S*-PTZ and *S*-DPZ could be attributed to incomplete conversion of S-DPZ. *S*-TBZ was found to be the more active enantiomer with 15-fold lower MIC_50_ than *R*-TBZ. Both enantiomers of TBZ and DTZ bind in the active site via azole coordination to the heme iron, with the tertiary hydroxyl on the central quaternary carbon oriented in the direction towards Y140. Like FLC, water 743 was present in both DPZ structures but was not detected in the TBZ structures, possibly due to the lower resolution of the crystal structure obtained. *S*-TBZ binds with its phenylethyl substituent anchored deep in the hydrophobic pocket. The *R* enantiomer presents the flexible phenylethyl substituent into the more open cavity and is subsequently disordered (data in S1 Fig). Conversely the shorter and more rigid chlorobenzyl substituent of *S*-DPZ appears to be an optimal fit for this cavity, with a close contact between the 2-chloro substituent and F236. The same 2-chloro substituent on the *R* enantiomer is also close to F236 but in a different orientation which pushes the chlorocyclopropyl group close to Y126. Overall this makes *S*-DPZ the more optimal fit.

The triazole fungicide DFC was first introduced in 1989 as a mixture of four stereoisomers. It was found to be the second most potent compound behind *S*-DPZ tested in this study against yeast. The crystal structure reveals all four stereoisomers bind in the same orientation, with three stereoisomers showing equivalent occupancy. The potency of DFC when compared with *S*-TBZ and *S*-DPZ can be attributed to deep binding of the phenoxyphenyl group in combination with the 1,3-dioxalane ring. A study of the stereoselective bioactivity of DFC against 4 different fungal pathogens (*Alternaria sonali*, *Fulvia fulva*, *Botrytis cinerea* and *Rhizoctonia solani*) identified the 2*S*,4*R* stereoisomer (5LZ; our nomenclature) as the most active compound [30]. The methyl group of this isomer projects towards Y126 (away from the heme) and would therefore be the most optimal fit of the four compounds.

Numerous amino acid mutations and deletions have been associated with reduced azole susceptibility in CYP51 in the phytopathogen *Z*. *tritici* (*M*. *graminicola*) [31] as well as the human pathogens *Candida albicans* and *Aspergillus fumigatus* [5, 19, 32]. Mutation of tyrosine 140 (Y140, *S*. *cerevisiae* numbering) is commonly found in many fungal species. For example, Y137F in *Z*. *tritici* confers resistance to triadimenol, Y132F/H confer resistance to FLC and VCZ in *C*. *albicans*, Y132H in combination with I471T confers enhanced azole resistance to FLC and VCZ in the *C*. *albicans* Darlington strain [33], and TR46/Y121F/T289A in *A*. *fumigatus* CYP51A, which is thought to have arisen due to agricultural use of azoles [5, 6], confers resistance to VCZ. Other mutations have been associated with reduced susceptibility to azole agrochemicals. For example, *Z*. *tritici* I381V confers resistance to TBZ [34] and *Z*. *tritici* V136A confers resistance to PRZ [35]. The structures presented here help to clarify the positions of such mutations relative to the bound ligands and justify susceptibility shifts seen though specific mutations. Residues V136, Y137 and I381 in *Z*. *tritici* Cyp51 (I139, Y140 and L380 in ScErg11p6×His) are all within the active site and project into the ligand binding cavity. Mutations associated with reduced sensitivity such as V136A (TBZ) and I381V (PRZ) induce an increase in the size of the binding pocket and lead to a less optimal fit for the ligand. Previous work has identified a key water mediated hydrogen bonding network between ScErg11p6×His Y140 and hydroxyl-containing azole inhibitors such as FLC and other inhibitors with structural similarities such as triadimenol and TBZ [22]. When mutations occur (either to phenylalanine or histidine) this hydrogen bonding network is disrupted and binding affinity decreases leading to reduced susceptibility. The cell based data presented here also shows reduced susceptibility to azole compounds in Y140F and Y140H mutant strains particularly when a hydroxyl group is present. As none of the compounds tested in the present study have a tail comparable to ITC or PCZ that penetrates deep into the substrate entry channel, the effects on affinity imposed by mutations at Y140F/H are not compensated for. The deletions ΔY459/G460 in *Z*. *tritici* CYP51 (corresponding to Y447/G448 in ScErg11p6×His), in conjunction with other mutations, confers resistance to TBZ and epoxiconazole [35, 36]. The homologous residues in ScErg11p6×His are situated at a turn at the end of the fungal specific loop region which is positioned on the opposite side of the heme cofactor well away from the ligand binding site. How this deletion impacts resistance from its distant position remains unclear.

Evidence has emerged [12, 13] around the development of cross-resistance in *Candida glabrata* [37] and *Aspergillus fumigatus* [38] to medically used triazoles as a result of agricultural compounds. This signals an urgent need to develop new fungicides to overcome resistance. The results presented allow for a clear understanding of the how the agrochemical fungicides act at their molecular target and reveal how mutations can affect drug binding. Until we able to achieve molecular resolution of CYP51s from plant pathogens, the yeast model provides a highly informative platform to investigate ligand binding, the impact of these mutations and a template for molecular models that will aid the discovery of next-generation antifungals for medical and agrochemical intervention.

## Materials and Methods

The plant pathogen inhibitors *R*-prothioconazole, *S*-prothioconazole, *R*-prothioconazole-desthio, *S*-prothioconazole-desthio, *R*-oxo-prothioconazole, *S*-oxo-prothioconazole, *R*-tebuconazole, *S*-tebuconazole, fluquinconazole and prochloraz were supplied by Bayer AG. Difenoconazole was purchased from Sigma-Aldrich as a mixture of four stereoisomers.

### Yeast strains overexpressing wild type and mutant ScErg11p6×His

The yeast strains used in this study have been reported previously [20–22, 25] and are described in S2 table.

### Phytopathogen CYP51 inhibitor susceptibility of strains overexpressing ScErg11p

The susceptibilities of strains overexpressing wild type ScErg11p6×His and ScErg11p6×His Y140F/H to CYP51 inhibitors were measured as MIC_50_ values using broth microdilution assays as described previously.[21] The MIC_50_s were defined as the concentration of drug required for 50% growth inhibition compared to no drug controls.

### Protein expression and purification

ScErg11p6×His expression and purification was carried out according to the methods described previously [20, 21]. In brief, crude membranes were prepared from liquid cultures grown overnight in YPD medium (1% (wt/vol) yeast extract (BD Difco^™^), 2% (wt/vol) peptone (BD Difco^™^), 2% (wt/vol) dextrose) at 30°C with shaking at 200 rpm. Cells were harvested and broken using bead beating and crude membranes were obtained by differential centrifugation. These crude membranes (5 mg/ml) were solubilized with 10× critical micelle concentration (CMC) *n*-decyl-β-D-maltoside (DM, Affymetrix Inc., Santa Clara, US). ScErg11p6×His was purified from the solubilized crude membrane fraction by affinity chromatography using 2 ml of packed Ni-NTA-agarose matrix (Qiagen) per 1 g of protein. Affinity purification buffer containing 10% (wt/vol) glycerol, 250 mM NaCl, 20 mM Tris pH 7.5, 0.5 mM PMSF, 16 mM (10×CMC) DM, 20 mM imidazole and 1 EDTA-free protease inhibitor pill per 200 mL. The affinity purified ScErg11p6×His was concentrated by centrifugal filtration using a 50 kDa molecular-weight cut-off Amicon Ultra-4 centrifugal filter (Millipore) and further purified by size exclusion chromatography (SEC) using a Superdex^™^ 200 10/300 GL column (GE Healthcare Life Sciences, UK). The column was equilibrated with SEC buffer containing 10% (wt/vol) glycerol, 250 mM NaCl, 20 mM Tris, pH 7.5, 0.5 mM phenylmethanesulfonyl fluoride, 6.4 mM DM (4×CMC) and 1 Roche EDTA-free protease inhibitor pill per 200 mL. CYP51 inhibitors dissolved in dimethyl sulfoxide (DMSO) were added to the pooled fractions obtained by size-exclusion chromatography with final concentrations of 40 μM to 1% of the final volume. The samples were then concentrated using a 50 kDa molecular-weight cut-off Amicon Ultra-4 centrifugal filter prior to crystallisation.

### Crystallization and X-ray data collection

Ni-NTA-agarose affinity and SEC purified ScErg11p6×His was concentrated and co-crystallized with ligands using the hanging-drop vapour-diffusion method (30). Crystals formed within one week at 18°C. Reservoir solutions contained 45% polyethylene glycol-400 in 100 mM glycine at a pH range of 9.3–9.5. The drops were 4 μl in a 1:1 ratio of reservoir solution and ~20 mg/ml of the protein in SEC buffer. Crystals were flash-cooled in liquid nitrogen prior to storage and data collection. Single datasets were collected on the MX2 beamline at the Australian Synchrotron using an ADSC Quantum ADSC Quantum 315 detector. Data were indexed and integrated using iMosflm [39] and scaled with SCALA [40]. Molecular replacement was carried out using Phaser-MR [41] from Phenix [42] using ScErg11p6×His co-crystallized with lanosterol (PDB ID: 4LXJ) as template [20]. Refinement and modelling was performed using phenix.refine [42] and Coot [43] respectively. Geometric restraints for inhibitors were generated via the Grade Web Server (http://grade.globalphasing.org/cgi-bin/grade/server.cgi) from mol2 files created within SYBYL-X2.1.1. The inhibitors were modelled into the appropriate density in the active site and waters were added if at least one hydrogen bond was detected (2.5–3.3 Å).

### Type II binding CYP51 inhibitors to ScErg11p6×His

Samples of Ni-NTA-agarose affinity purified ScErg11p6×His were eluted with 40 mM histidine instead of imidazole as described by Warrilow *et al*. [44]. Samples were washed free of the histidine and concentrated by centrifugal filtration using a 50 kDa molecular-weight cut-off Amicon Ultra-4 centrifugal filter (Millipore) to give protein with a Soret peak at 417 nm. Carbon monoxide binding was used to determine the concentration of functional cytochrome P450 for drug binding studies as described previously [21] based on the protocol described by Guengerich *et al*. [45] Saturation curves for the binding of type II ligands were determined by obtaining difference spectra for functional enzyme at 1 μM as described previously [21]. The dissociation constant *K*_d_ for type II binding of CYP51 inhibitors was calculated by applying the Hill equation using the formula ΔA = ΔA_max_ [Azole]^*n*^ / ([Azole]^*n*^ + *K*_*d*_^*n*^), with ΔA_max_ being the maximum change in absorbance, [Azole] the azole concentration and *n* the Hill coefficient. All calculations were carried out using GraphPad Prism 6 Software (GraphPad Prism, San Diego, CA). [Azole]_0.5_ values were defined as the concentration of the azole drug that gave half ΔA_max_.

## Supporting Information

S1 Fig OMIT maps following initial refinement for (A) *S*-TBZ (green carbons), (B) *R*-TBC (cyan carbons), (C) *S*-DPZ (magenta carbons), (D) *R*-DPZ (salmon carbons), (E) FQZ (blue carbons), (F) PRZ (olive carbons), and (G) all four stereoisomers of DFC (purple carbons). Ligands are the final refined conformation. *Fo-Fc* map [green mesh] contoured at 3σ; 2*Fo-Fc* map [blue mesh] contoured at 1σ. Maps were calculated using *F*_*calc*_ refined from coordinates with no ligand at the active site. N atoms are coloured blue, Oxygen red, Chlorine green and Fluorine pale blue. The heme cofactor is shown as sticks with the iron atom (where visible) an orange sphere.
(PNG)

S2 Fig Difference spectra illustrating type II binding of (A) *S*-tebuconazole, (B) *R*-tebuconazole (C) *S*-prothioconazole-desthio (D) *R*-prothioconazole-desthio (E) Prochloraz (F) Fluquinconazole and the spectra for (G) *S*-oxo-prothioconazole and (H) *R*-oxo-prothioconazole indicating neither type I nor type II binding. The curves shown were obtained by incremental additions of the azole up to 2 μM, in the presence of 1 μM ScErg11p6×His. Representative examples of at least two experiments are shown.
(PNG)

S1 Table Data collection and refinement statistics. (DOCX)

S2 Table Yeast strains used in this study. (DOCX)

S3 Table Binding of azoles to affinity-purified ScErg11p6×His. (DOCX)
