# Supplementary figures and images for: Structural and Functional Elucidation of Yeast Lanosterol 14α-Demethylase in Complex with Agrochemical Antifungals
Source: PLoS One. 2016 Dec 1;11(12):e0167485. doi: 10.1371/journal.pone.0167485 (PMC5132298; doi:10.1371/journal.pone.0167485)

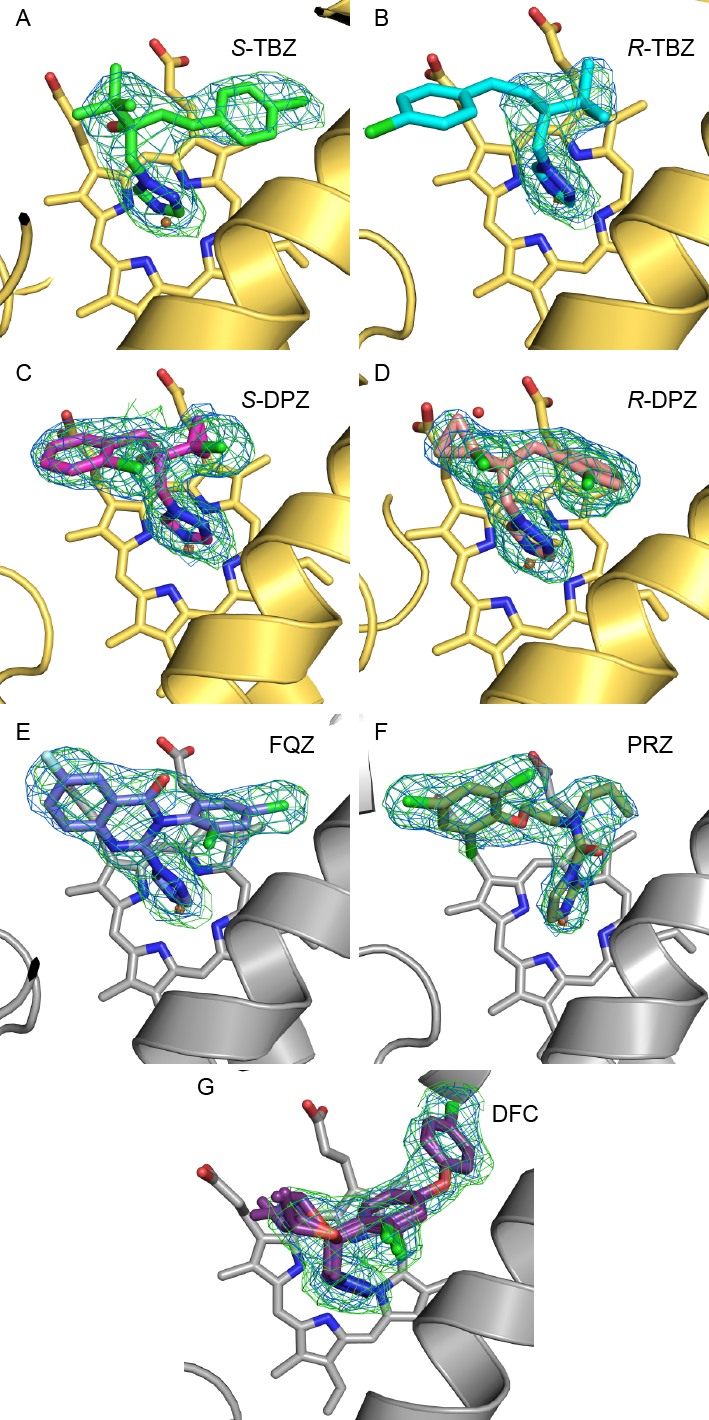

Supplement: S1 Fig — Ligands are the final refined conformation. Fo-Fc map [green mesh] contoured at 3σ; 2Fo-Fc map [blue mesh] contoured at 1σ. Maps were calculated using Fcalc refined from coordinates with no ligand at the active site. N atoms are coloured blue, Oxygen red, Chlorine green and Fluorine pale blue. The heme cofactor is shown as sticks with the iron atom (where visible) an orange sphere. (PNG) [file pone.0167485.s001.png]

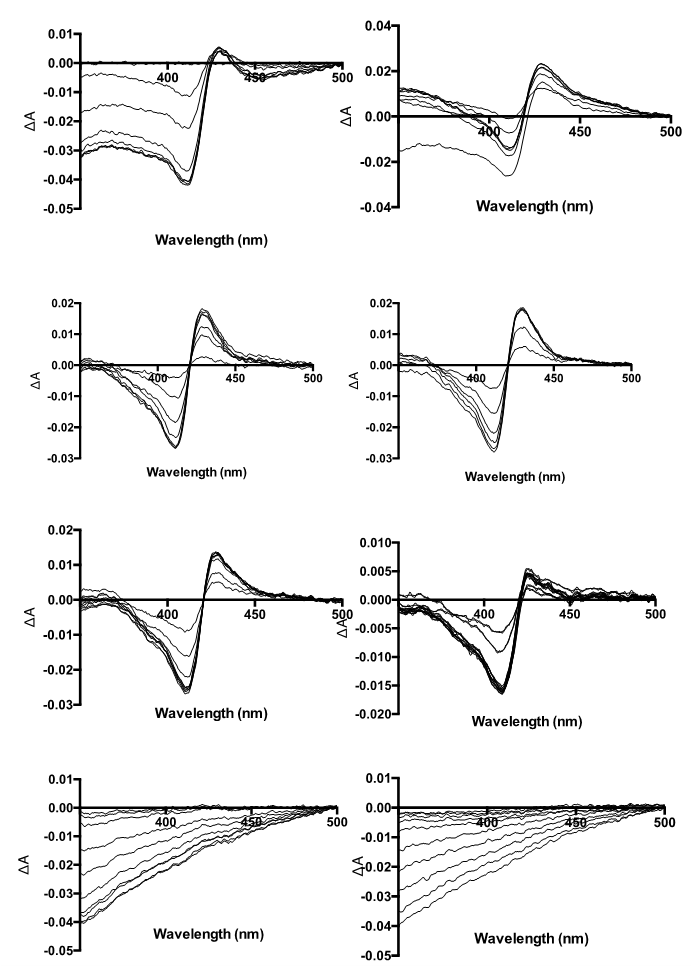

Supplement: S2 Fig — The curves shown were obtained by incremental additions of the azole up to 2 μM, in the presence of 1 μM ScErg11p6×His. Representative examples of at least two experiments are shown. (PNG) [file pone.0167485.s002.png]
