# Supplementary material for: Structural and Functional Elucidation of Yeast Lanosterol 14α-Demethylase in Complex with Agrochemical Antifungals
Source: PLoS One. 2016 Dec 1;11(12):e0167485. doi: 10.1371/journal.pone.0167485 (PMC5132298; doi:10.1371/journal.pone.0167485)
Supplement: S1 Table — (DOCX) [file pone.0167485.s003.docx]

**S1 Table** Data collection and refinement statistics.

Values in parentheses are for the highest resolution shell.

| PDB ID | 5EAB | 5EAC | 5EAD | 5EAE |
| --- | --- | --- | --- | --- |
| Inhibitor | *S*-Tebuconazole | *R*-Tebuconazole | *S*-Prothioconazole-desthio | *R*-Prothioconazole-desthio |
| Space group | P 1 2_1_ 1 | P 1 2_1_ 1 | P 1 2_1_ 1 | P 1 2_1_ 1 |
| Resolution range (Å) | 80.26 – 2.60 (2.72 – 2.60) | 29.94 - 2.26 (2.341 - 2.26) | 79.89 – 2.00 (2.071 - 2.0) | 79.95 - 2.05 (2.17 - 2.11) |
| Unit cell (Å) | a = 80.70 b = 67.23 c = 81.63 | a = 77.37, b = 67.33, c = 80.79 | a = 78.27, b = 67.18, c = 80.91 | a = 78.90, b = 67.68 c = 81.03 |
| Diffraction source | Australian Synchrotron MX2 | Australian Synchrotron MX2 | Australian Synchrotron MX2 | Australian Synchrotron MX2 |
| Wavelength (Å) | 0.954 | 0.954 | 0.954 | 0.954 |
| Total reflections | 102823 (12876) | 150826 (13443) | 219677 (16206) | 190026 (15896) |
| Unique reflections | 26558 (3242) | 38587 (3551) | 56012 (4131) | 48243 (3925) |
| Multiplicity | 3.9 (4.0) | 3.9 (3.8) | 3.9 (3.9) | 3.9 (4.0) |
| Completeness (%) | 99.5 (99.9) | 99.7 (99.3) | 99.6 (99.4) | 99.1 (99.0) |
| **Mean** (I/σ(I)) | 10.3 (2.1) | 9.7 (1.5) | 5.8 (1.4) | 8.2 (2.2) |
| R_merge_^†^ | 0.067 (0.578) | 0.079 (0.846) | 0.098 (0.609) | 0.107 (0.728) |
| CC(1/2) | 0.996 (0.864) | 0.997 (0.631) | 0.998 (0.923) | 0.988 (0.762) |
|  |  |  |  |  |
|  |  |  |  |  |
| **Refinement** |  |  |  |  |
| R_cryst_‡ | 0.2079 (0.3408) | 0.2042 (0.3227) | 0.1945 (0.3081) | 0.2023 (0.3303) |
| R_free_§ | 0.2628 (0.3660) | 0.2402 (0.3497) | 0.2250 (0.3437) | 0.2370 (0.3598) |
| **Number of atoms in model** |  |  |  |  |
| Protein | 4242 | 4287 | 4284 | 4281 |
| Ligand | 64 | 64 | 63 | 63 |
| Water molecules | 0 | 77 | 152 | 168 |
| Deviation from ideal bond lengths (Å) | 0.009 | 0.009 | 0.009 | 0.008 |
| Deviation from ideal bond angles (⁰) | 1.20 | 1.17 | 1.10 | 1.071 |
| **Ramachandran analysis (%)** |  |  |  |  |
| Preferred | 94 | 96 | 97 | 97 |
| Outliers | 0.57 | 0.57 | 0.38 | 0.19 |

† R_merge_ = Σ*_hkl_* Σ*_i_* |*I_i_(hkl)* – [*I(hkl)*]| / Σ*_hkl_* _i_ *I_i_*(*hkl*).

‡ R_cryst_ = Σ_hkl_ |F_obs_-F_calc_|/ Σ_hkl_ |Fobs| computed over a working set composed of 95% of data.

§ R_free_ = Σ_hkl_ |F_obs_|-|F_calc_| / Σ_hkl_ |F_obs_| computed over a test set composed of 5% of data.

| PDB ID | 5EAF | | 5EAG | 5EAH |
| --- | --- | --- | --- | --- |
| Inhibitor | Fluquinconazole | | Prochloraz | Difenoconazole |
| Space group | P 1 | | P 1 2_1_ 1 | P 1 2_1_ 1 |
| Resolution range (Å) | 29.9 - 2.65 (2.745 - 2.65) | | 29.63 - 2.4 (2.486 - 2.4) | 79.99 - 2.54 (2.65 - 2.54) |
| Unit cell (Å) | 67.56 79.6 81.13 | | 79.44 67.89 81.77 | 78.02 67.33 80.90 |
| Diffraction source | Australian Synchrotron MX2 | | Australian Synchrotron MX2 | Australian Synchrotron MX2 |
| Wavelength (Å) | 0.954 | | 0.954 | 0.954 |
| Total reflections | 88236 (11415) | | 120477 (12982) | 86668 (10014) |
| Unique reflections | 24657 (3238) | | 33221 (3509) | 27403 (3320) |
| Multiplicity | 3.6 (3.5) | | 3.6 (3.7) | 3.2 (3.0) |
| Completeness (%) | 99.2 (0.867) | | 98.6 (99.5) | 99.3 (99.0) |
| **Mean** (I/σ(I)) | 6.8 (1.5) | | 5.3 (1.6) | 6.6 (1.8) |
| R_merge_^†^ | 0.101 (0.625) | | 0.101 (0.473) | 0.103 (0.768) |
| CC(1/2) | 0.992 (0.867) | | 0.992 (0.923) | 0.984 (0.521) |
| **Refinement** |  | |  |  |
| R_cryst_‡ | 0.2094 (0.3123) |  | 0.1988 (0.2784) | 0.1954 (0.2724) |
| R_free_§ | 0.2580 (0.3922) |  | 0.2519 (0.3627) | 0.2505 (0.3145) |
| **Number of atoms in model** |  | |  |  |
| Protein | 8540 | | 4271 | 4277 |
| Ligand | 136 | | 66 | 151 |
| Water molecules | 36 | | 126 | 37 |
| Deviation from ideal bond lengths (Å) | 0.010 | | 0.009 | 0.008 |
| Deviation from ideal bond angles (⁰) | 1.35 | | 1.21 | 1.02 |
| **Ramachandran analysis (%)** |  | |  |  |
| Preferred | 96 | | 95 | 95 |
| Outliers | 0.29 | | 0.57 | 1.7 |
