# Supplementary material for: Structural and Functional Elucidation of Yeast Lanosterol 14α-Demethylase in Complex with Agrochemical Antifungals
Source: PLoS One. 2016 Dec 1;11(12):e0167485. doi: 10.1371/journal.pone.0167485 (PMC5132298; doi:10.1371/journal.pone.0167485)
Supplement: S2 Table — (DOCX) [file pone.0167485.s004.docx]

**S2 Table. Yeast strains used in this study**

| Strain | Genotype | Reference |
| --- | --- | --- |
|  |  |  |
| AD2Δ | MATα *PDR1-3 Δyor1::hisG Δsnq2::hisG Δpdr3::hisG Δpdr10::hisG Δpdr11::hisG Δycf1::hisG Δpdr15::hisG Δpdr5*::*hisG*  *Δura3::200 Δhis1::dpl200* | (1) |
| AD3Δ | AD2Δ *Δerg11::HIS1* | (1) |
| AD3Δ_Y140F | AD3Δ *Δpdr5::ScErg11p*_Y140F | (1) |
| AD3Δ_Y140H | AD3Δ *Δpdr5::ScErg11p*_Y140H | (2) |
|  |  |  |

**References**

1. **Sagatova AA, Keniya MV, Wilson RK, Monk BC, Tyndall JD.** 2015. Structural Insights into Binding of the Antifungal Drug Fluconazole to *Saccharomyces cerevisiae* Lanosterol 14alpha-Demethylase. Antimicrob Agents Chemother **59:**4982-4989.

2. **Sagatova AA, Keniya MV, Wilson RK, Sabherwal M, Tyndall JD, Monk BC.** 2016. Triazole resistance mediated by mutations of a conserved active site tyrosine in fungal lanosterol 14alpha-demethylase. Sci Rep **6:**26213.
