# Supplementary material for: Structural and Functional Elucidation of Yeast Lanosterol 14α-Demethylase in Complex with Agrochemical Antifungals
Source: PLoS One. 2016 Dec 1;11(12):e0167485. doi: 10.1371/journal.pone.0167485 (PMC5132298; doi:10.1371/journal.pone.0167485)
Supplement: S3 Table — (DOCX) [file pone.0167485.s005.docx]

**S3 Table. Binding of azoles to affinity-purified ScErg11p6×His**

| Compound | Induced spectral shift on ligand binding  Wavelength (nm) | [Azole]_0.5_  (µM) | Hill coefficient (n) | K_d_ (nM) ± SE |
| --- | --- | --- | --- | --- |
| *S*-Tebuconazole | 417 → 421 | 0.33 | 2.5 | 115 ± 44 |
| *R*-Tebuconazole | 417 → 421 | 0.30 | 2.0 | 65 ± 35 |
| *S*-Prothioconazole | 417 (-) | - | - | - |
| *R*-Prothioconazole | 417 (-) | - | - | - |
| *S*-Prothioconazole-desthio | 417 → 422 | 0.42 | 2.8 | 102 ± 45 |
| *R*-Prothioconazole-desthio | 417 → 422 | 0.33 | 2.3 | 54 ± 31 |
| *S*-Oxo-prothioconazole | 417(-) | - | - | - |
| *R*-Oxo-prothioconazole | 417(-) | - | - | - |
| Fluquinconazole | 417 → 419 | 0.31 | 2.5 | 15 ± 21 |
| Prochloraz | 417 → 421 | 0.30 | 2.1 | 27 ± 24 |

Type II binding curves were obtained by incremental additions of the azole to 1 µM ScErg11p6×His (Fig S2). Hill coefficients and K_d_ values were obtained by applying the Hill equation as described in Materials and methods. (-) indicates no significant change in Soret peak wavelength on exposure to the ligand. The data presented is a representative set
